# Characterization of mAb aggregates in a mammalian cell culture production process

**DOI:** 10.1186/1753-6561-7-S6-P80

**Published:** 2013-12-04

**Authors:** Albert Paul, Friedemann Hesse

**Affiliations:** 1 Institute of Applied Biotechnology, University of Applied Sciences Biberach, Biberach, 88400, Germany

## Introduction

Protein aggregation is a major concern during monoclonal antibody (mAb) production [1,2]. The presence of aggregates can reduce the therapeutic efficacy of mAbs and trigger immunogenic responses upon administration [3]. Higher molecular weight (HMW) aggregates can be removed during downstream processing (DSP), but prevention of aggregate formation upstream could increase process yield [4,5]. Unfortunately, detection of aggregates upstream is challenging, since the size of aggregates ranges from small oligomers to visible particles and there is no single technique capable of measuring the broad range of aggregation phenomena [6,7]. For upstream detection of aggregates, all HMW species potentially present in the culture broth must be known. Therefore, we established methods to generate different types of aggregates and characterized the different HMW species using size exclusion high pressure liquid chromatography (SE-HPLC), dynamic light scattering (DLS) and UV spectroscopy. Furthermore, stability and traceability of the aggregates in cell culture medium and Chinese hamster ovary (CHO) DG44 supernatant were demonstrated. Finally, the established methods were used to monitor aggregate formation in a mAb producing CHO DG44 cell culture.

## Material and methods

Two mAbs produced in CHO DG44 cells and stored in 20 mM acetate at pH 3.5 were used for aggregation studies. Aggregation was induced using heat stress, pH-shift, high salt concentration and freeze-thawing. Heat stress was induced at 65 °C for different time periods. For the pH-shift, the antibody was diluted in citrate-phosphate buffer containing pH 3-8. NaCl concentrations for salt-induced aggregation varied from 50-1500 mM. A freeze-thawing cycle included incubation at -80 °C for 15 min followed by thawing at 25 °C for 15 min. The freeze-thaw cycle was repeated three times. The presence of small aggregates was evaluated using SE-HPLC equipped with a Yarra SEC4000 (Phenomenex) column. To identify the different HMW species the molecular weight was determined using SEC-MALS (multi-angle light scattering). Moreover, large aggregates were characterized using DLS (Zetasizer 3000HS, Malvern instruments) and UV spectroscopy (SpectraMax M5^e ^microplate reader, Molecular Devices). The size of large aggregates was displayed by the average diameter. The aggregation index (AI) was calculated from UV absorbance using the following equation: A_340_×100/(A_280_-A_340_). Furthermore, stability of induced aggregates in cell culture medium (SFM4CHO, Thermo Scientific) and CHO DG44 host cell supernatant was investigated. Therefore, freeze-thawed mAb2 was spiked into the culture medium as well as CHO DG44 host cell supernatant and analyzed via SE-HPLC. Finally, the supernatant of CHO DG44 mAb2 producer cells was analyzed directly after inoculation and at the end of cultivation. Based on results obtained from spiking aggregated mAb2 into CHO DG44 host cell supernatant, aggregate formation in a culture of a mAb producing CHO DG44 cell line was monitored.

## Results

All stress methods provoked aggregate formation. The mAbs showed formation of different aggregates using the different stress methods (Table 1). Heating the antibody only led to formation of large aggregates. Despite the loss of mAb2 monomer, no small aggregates were detected via SE-HPLC. However, heat induction provoked formation of large aggregates, whereby the average size (diameter > 1 μm) and AI increased over time at 65 °C. Hence, heat induction can only be used to generate large aggregates of the mAbs used in this study. The pH change provoked formation of small aggregates (dimer and oligomer) as well as large aggregates (diameter > 75 nm). With increasing pH dimer and oligomer levels also increased, whereas an increased diameter was only observed for pH 5 and 6. Thus, a shift to pH 6 can be used for induction of dimers, oligomers and large aggregates. The addition of NaCl provoked concentration-dependent formation of dimers and large aggregates (diameter > 50 nm) at higher NaCl concentrations (above 500 mM). In contrast to pH-induction, no oligomers larger than dimer were visible via SE-HPLC. Therefore, NaCl can be used for the fast generation of dimers and above a concentration of 500 mM for the induction of large aggregates. With increasing freeze thaw cycles formation of small aggregates occurred. Surprisingly, more aggregates were formed than with all other methods. Hence, freeze-thawing was used to study the stability of aggregates under culture conditions.

**Table 1:** Formation of different HMW species using different induction methods.

Induction Method	Small aggregates		Large aggregates
	***Dimer***	***Oligomer***	
Heat	-	-	Up to 1 μm
pH	Increase with pH	+	At pH 5 and 6
NaCl	Increase with NaCl	-	Above 500 mM
Freeze-thawing	+	+	-

**Figure 1:**
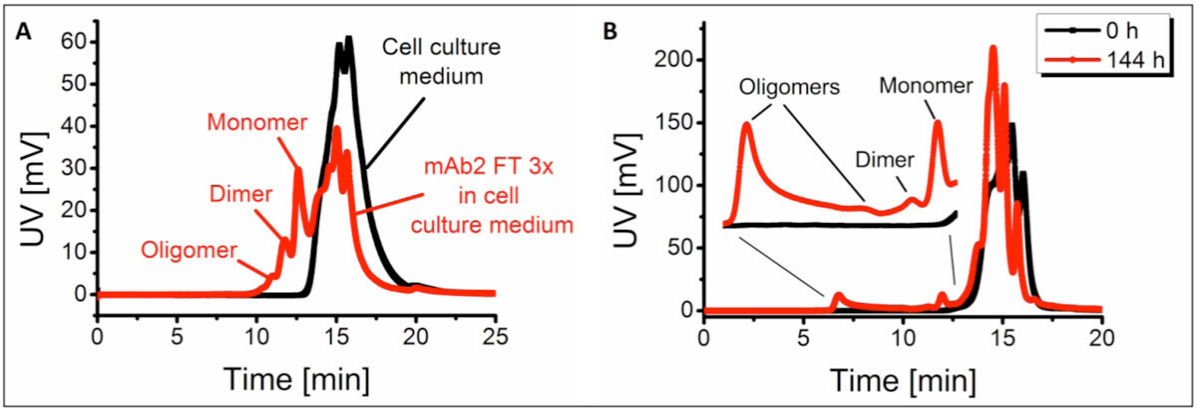
**Freeze-thawed mAb2 spiked into cell culture medium (A) and supernatant of CHO DG44 mAb producer cells (B)**.

For this purpose, freeze-thawed mAb2 was spiked into culture medium and analyzed using SE-HPLC (Figure 1, A). Since the retention time of cell culture medium components differed from the freeze-thawed antibody, monomer and the aggregates were still detectable. Accordingly, freeze-thawing was preferred for the use in cell culture supernatant spiking experiments. The investigation of freeze-thawed (3x) mAb2 spiked into CHO DG44 host cell supernatant revealed that mAb2 monomers as well as the aggregates (22% dimer and 72% oligomer) were still detectable and quantifiable via SE-HPLC. Knowing the retention time of different aggregate species, the analysis of the supernatant of a CHO DG44 mAb producing cell line was performed at the beginning and after 144 h cultivation (Figure 1, B). Aggregates and monomer could successfully be detected after 144 h via SE-HPLC, whereas after inoculation neither monomer nor aggregates were visible. Therefore, the methods established in this work can be used to generate different types of aggregates as positive control to evaluate aggregate formation in cell culture supernatant.

## Summary

The stress methods used in this work induced different types of aggregates. Heating the antibody led to a loss of monomer and only formation of large aggregates. Dimers and oligomers were formed with increasing pH and large aggregates were formed at pH 5 and 6. A NaCl concentration dependent aggregate formation was observed, whereby only dimers were visible via SE-HPLC and large aggregates were only present at a NaCl concentration above 500 mM. Freeze-thawing induced more aggregates as with all other methods and was therefore used for the application under cell culture conditions. Spiking experiments of freeze-thawed mAb2 in culture medium and CHO DG44 host cell supernatant revealed that aggregates were still detectable and quantifiable under cell culture conditions. Finally, this work showed that aggregate formation directly in the supernatant of a CHO DG44 mAb producing cell line is possible.
